# Work related cardiovascular load in professional dance teachers – a pilot study

**DOI:** 10.1186/s12995-020-00257-0

**Published:** 2020-03-23

**Authors:** Eileen M. Wanke, Mike Schmidt, Gerhard Oremek, David A. Groneberg

**Affiliations:** 1 Johann Wolfgang von Goethe University, Institute of Occupational Medicine, Social Medicine and Environmental Medicine, Theodor-Stern-Kai 7, 60590 Frankfurt am Main, Germany; 2 University of Hamburg, Department of Sports and Exercise Medicine, Institute of Human Movement Science, Mollerstrasse 10, 20148 Hamburg, Germany

**Keywords:** Physical effort, Work, Dance teacher

## Abstract

**Objective:**

There have been only a limited number of studies available on the physical requirements in dance teachers (DT), who are responsible for the training of recreational and/or professional dancers and/or dance students. First results provide indications that a consideration of physical work load (teaching load) of this occupational group is necessary.

**Methods:**

HR measurements were done on a total of 21 DT (f: *n* = 18, m: *n* = 3) aged 48.2 ± 9.3 years during their lessons in three dance styles: ballet (B), jazz/modern dance (J/MD) and pre-school dance (CD). The HR data were objectified using the spiroergometrically measured maximum HR (HRmax). In addition, the rating of perceived exertion (RPE) was asked directly after the lesson using the Borg scale together with an additional questionnaire.

**Results:**

Depending on the dance style, the average HR load during the lessons ranged between 56.7% ± 7.4%) (B) and 63.6% ± 9.8% (CD) of the individual HRmax. No significant differences could be found between the dance styles for the minimum, medium and maximum teaching loads. The DTs rated the average RPE of the teaching units according to BORG (11.4 ± 2.1). Correlations between the RPE and the mean and maximum cardiovascular loads (r = 0.376, *p* = 0.037 and r = 0.441, *p* = 0.013) could be shown for all dance units and for the mean loads in J/MD (r = 0.558, *p* = 0.044).

**Conclusion:**

Most dance units do not represent a load in the sense of a endurance training unit. An increase in aerobic fitness and possible positive effects in the context of injury prevention is not to be expected. In J/MD, the use of RPEs for the rough estimation of cardiovascular stress is conceivable. The assessment of cardiovascular load in dance lessons requires further investigations for more precise assessments.

## Background

The main task of a dance teacher is to teach a specific dance technique in combination with a musical and artistic understanding based on pedagogical and social competence [1, 2]. That means in detail that, besides their role model function, they are responsible for the continuity and further development of dance techniques as well as the choreographic quality. The degree of the subjectively perceived physical and psycho-mental workload strongly depends on the target group and the performance standard [3, 4]. For advanced and professional dancers indicated movement sequences or a pure verbalization of the sequence of exercises are sufficient, while for beginners and children the entire movement sequences are to be physically demonstrated several times [5].

Apart from the musical accompaniment, e.g. the barre or the footwear, there are only few tools to facilitate the working process available for dance teachers. Hence the workloads have a direct effect on their body. Keeping one’s own body healthy is therefore of existential importance in dance pedagogy, especially since the majority (82.4%) of dance teachers work on a freelance basis. As it is difficult to compensate for absenteeism, 89.4% of them continue to work even in pain [4, 6].

### Objective of the present study

Despite the importance of the body and the popularity of dance, objective data on workloads are still very limited or are of earlier date [4, 5]. On the basis of own estimations, initial results suggest that physical demands can be high to maximum [4] and therefore require a closer consideration with more objective methods. The initial results showed cardiovascular loads reaching to the sub-maximal range and thus point to a further need for research. The aim of this study is to illustrate the cardiovascular stress on the basis of heart rates caused by teaching using the example of three popular dance styles. It can be assumed that there are differences between the dance styles [3]. Possible differences between the three dance styles in the cardiovascular load profiles are highlighted and minimum, average and maximum loads are compared.

In this context it will also be assessed whether dance units could be a training stimulus to increase basic stamina or whether additional compensatory training would be necessary.

Earlier results showed significant fluctuations in the range of mean to submaximal cardiovascular loads [3]. It can be assumed that not all teaching units have a endurance enhancing character, but only short-term peak loads. Twitchett et al. (2010a) [7] have already pointed out, that at the end of a dance-specific fitness test the heart rate in ballet students is positively associated with the occurrence of injuries as an indicator of poorer aerobic fitness. Conversely, this would mean that higher cardiovascular fitness would result in less injuries and therefore less absenteeism.

In addition, the assessment of the subjective state of exhaustion by means of RPE is intended to test a possible instrument for self-assessment of cardiovascular hourly load, since this could be easily used in the context of behavioural changes.

## Methods

### Subjects

The inclusion and exclusion criteria are shown in Table 1. Recruitment was started by the German Dance Teacher Association (DBfT) and the Royal Academy of Dance (RAD) through a call for voluntary work-related preventive examinations. The appeal was addressed to all dance teachers in Berlin. In addition, the study information was sent to larger Berlin dance schools and passed on to well-known teachers. The volunteers were recruited and examined within a period of 2 months. This included the measurements during their dance units. As a pilot project, the sample size is based on the number of volunteers available in the above mentioned period. Not all dance teachers were teaching in all three dance styles. A first appointment took then place at the workplace (dance school). Participation in the study was voluntary and free of charge. The investigations followed ethical research criteria. Study procedures were approved by the University Research Ethics Committee (Charité University – Medical School, Berlin, Germany).

**Table 1:** Inclusion and exclusion criteria

Inclusion criteria	Exclusion criteria
- at least 20 years old - full-time activity as a dance instructor - Lessons in at least one of the following dance styles: classical dance, modern dance, jazz dance, creative children’s dance/early dance education - employed or freelance - at least 10 lessons per week - at least 20 h of total working time per week	- exclusively teaching professional dancers - acute or chronic injuries, and/or illnesses contradicting the typical teaching load - Failing to meet the inclusion criteria - Long-term cardiovascular medication

### Recording of the cardiorespiratory teaching load (heart rate monitoring)

Two heart rate monitors (receivers) and a transmitter belt (S625X from Polar) were used to record the heart rate during one to two dance units. The transmitter-receiver system was set to register the heart rates at 5-s intervals and store them in the receivers. Afterwards the values were read out via the Polar software (Polar ProTrainer 5 version 5.41.002) and the raw values were exported for further data processing. The system was activated immediately prior to each training unit and deactivated at the end.

An electronic observation protocol was written during the entire unit documenting general data on location, time, teaching content, target group and dance style. The three popular dance styles selected for comparison were classical dance (ballet), contemporary (jazz dance/modern) dance as well as early dance education, also called creative dance or children’s dance.

### Ratings of perceived exertion (according to BORG)

The scale for ratings of perceived exertion (RPE scale) developed by Borg (1982) [8] was used to assess the subjective perception of physical exertion. Okura & Tanaka (2001) [9] showed that Borg’s RPE scale is suitable to provide a good prognosis for VO2max (r = 0.781) and oxygen uptake at the anaerobic threshold (VO2at; r = 0.790). In sports, it is mainly used to evaluate training loads and to control processes [10–14]. It is generally considered a reliable and valid measuring instrument [10] in dance [15].

### Questionnaire on professional activities

A brief version of a questionnaire for DT developed by Wanke et al. (2015) [4] was used. It served as an initial assessment of the professional situation and collected general data on personal characteristics and full-time employment.

### Testing procedure

The tests were carried out at the work places (e.g. dance schools) of the dance teachers. As agreed upon, each dance unit was a typical average lesson meeting the following criteria:

- DT and students have already completed at least 8 lessons together
- performance standard is at advanced I and advanced foundation level (Royal Academy of Dance)
- focus of the lesson is the teaching of techniques (neither choreography nor rehearsals)
- at least 50% of the students of the core group are present
- a maximum break of 2 weeks is allowed between the last lesson and the test unit
- at least two more lessons are to follow in the 2 weeks after the test unit (as a criteria for regular teaching)
- Dts on long-term cardiovascular drugs that could alter the heart rate were excluded (e. g. beta blocker, cardiac glycosides)
- All ballet classes contained barre as well as center work (common with advanced foundation und advanced I)

In addition, the DT was instructed to give a teaching unit comparable with the lessons of the past weeks in terms of exercise contents and lesson structure. Prior to each lesson, a brief description was given of how the measurement is performed. Immediately afterwards, the test subjects were asked to estimate the average physical effort during the teaching units by means of the RPE (ranging from 6 = ‘very, very easy’ to 20 = ‘very very hard’) [8] as an average for the teaching unit.

### Data analysis

Due to the low (*n* = 3) proportion of male DT, a gender-specific evaluation of the volunteers was waived.

Microsoft Excel 2010 was used for data preparation with IBM SPSS 19 being used for analytical statistics. The raw heart rate protocols recorded by the heart rate monitors were imported into Excel. Then the time course was compared with the movement protocols generated in the hours. The beginning of the lesson was defined as the time of the verbal greeting and the end as the verbal farewell by the dance teacher.

The representation of the heart rate during the lesson was made after an average curve was generated from the individual lessons and the calculation of the associated standard error. In order to allow more precise statements about differences in the course of cardiovascular stress between the three dance styles mentioned, the lessons were divided into two sections, the warm-up phase (T1, for example warm-up phase in J/MD, set-up phase in pre-school dance and barre training in B) and a main phase (T2, center work after warm-up phase) according to the specifications of Dahlström (1997) [3] on the basis of Cohen et al. (1982) [16] and Schantz & Astrand (1984) [17]. The absolute heart rates of each subject were relativized to the individual maximum heart rate and expressed as % HRmax to objectify the heart rates for the comparison between both the dance styles and the lesson sections. Prior to that, the maximum functional capacity (VO2peak) and maximum heart rate (HRpeak) had been determined by spiroergometry. Female TPs completed a gradual exercise on the bike ergometer starting at 80 W (or 100 W for their male colleagues) and a continuous increase of 40 W every three minutes (or 50 W for the men).

### Statistics

Information on the descriptive statistics of the sample was provided by presenting the mean values and the associated standard deviations. All metric data were checked for normal distribution using the Shapiro-Wilk test and for homogeneity using the Levene test. The significance level was set at *p* = 0.05 for bilateral questioning.

The comparison of the minimum, medium and maximum heart rates between the dance styles was made using the single factor Anova and the Fischer LSD post hoc test. In case inhomogeneity existed in one or more parameters, the Kruskal-Wallis test was used alternatively.

Regarding the lesson sequences T1 and T2 in the three dance styles, the variance analysis with measurement repetition and the LSD post hoc test were used to determine effects on the time, between the groups and the interaction.

The linear correlation between the physical load based on the relative heart rate (% HRmax) and the RPE were checked using the Spearman correlation coefficient. In case of positive linear correlations a regression equation was determined.

## Results

Table 2 shows the anthropometric characteristics of the sample collected. It should be emphasized that the sample consisted of healthy, middle-aged female test subjects with a standard body according to BMI. The weekly working hours and the high number of years of professional experience support experienced DT in a main profession. The spiroergometric measurements showed an average capacity of 165.0 W (SD: 44.4 W), a body weight-related capacity of 2.7 W/kg (SD: 0.6 W/kg) and an average maximum oxygen uptake of 29.5 ml/kg*min (SD: 7.1 ml/kg*min).

**Table 2:** Anthropometric data of the sample mean values (mean value ± standard deviation)

**Gender**	**N (%)**
Female	18 (85.7)
Male	3 (14.3)
**Descriptive data**	**Mean value ± SD (*n*** **= 21)**
Age (a)	48.2 ± 9.3
Height (cm)	169.5 ± 7.1
Weight (kg)	61.1 ± 8.8
BMI (kg/m^2^)	21.2 ± 2.3
Care years (a)	18.3 ± 10.3
Lessons per week (h/7d)	17.3 ± 4.8

### Heart rates during the lessons

**Fig. 1:**
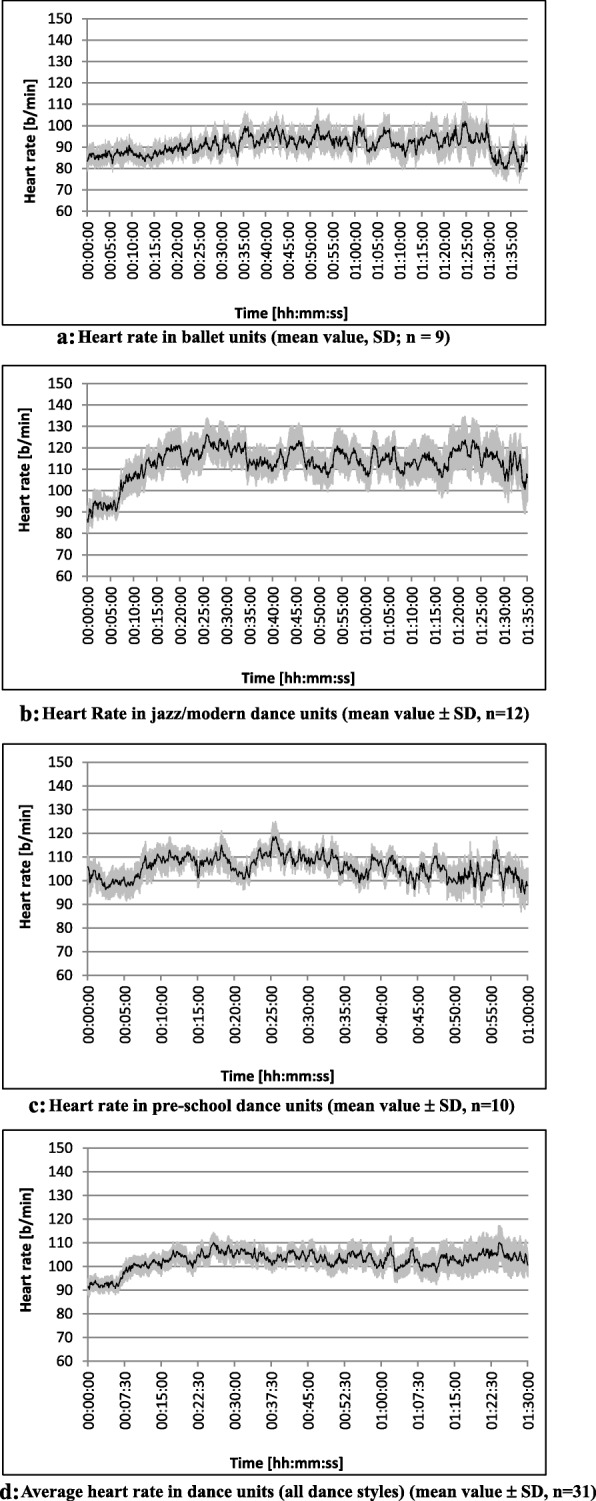
**a** Heart rate in ballet units (mean value, SD; n = 9). **b**: Heart Rate in jazz/modern dance units (mean value ± SD, n = 12). **c**: Heart rate in pre-school dance units (mean value ± SD, n = 10). **d**: Average heart rate in all dance styles (mean value ± SD, n = 31). Pre- school dance (*n* = 9)

Figure 1a-d show typical heart rates in the different dance styles. In the ballet lesson it became apparent that the heart rate fluctuated slightly - usually less than 10 beats/min in the first third of the lesson. Only in the second and third a more frequent change of low and higher heart rates with longer peaks was observed. The differences were mostly between 10 and 15 beats (Fig. 1a). In general, the initial frequencies were repeatedly reached throughout the entire lesson. The heart rates ranged between 85 and 100 beats/min.

In J/MD, a much clearer increase of about 30 beats/min was recorded from the 7th to the 20th min. Thereafter, higher and lower load plateaus alternated. The differences between these ranged between 10 and 15 beats/min for an approximate duration of 3 to 5 min with the frequency range between 110 and 125 beats/min (Fig. 1b).

The pre-school dance was characterized by more frequent heart rate fluctuations of 10 beats/min, usually recognizable as peak loads with a duration of 30 to 90 s. Throughout the course of the lesson, the heart rate repeatedly dropped to the initial values of about 100 beats/min. In the middle of the lesson (between the 17th and 25th min) utmost fluctuations of up to 20 beats/min could be observed (Fig. 1c).

Considering a characteristic dance lesson of each of the three dance styles, the average heart rate was relatively constant with numerous peaks in the range of 10 beats/min. Heart rates average ranged from 110 to 120 beats/min during the entire lesson (Fig. 1d). The standard errors (grey markings in Fig. 1a-d) turned out slightly lower in the pre-school dance than in B and J/MD. It can be assumed that there are major individual differences between individual lessons, especially in J/MD and the middle part of the ballet lessons.

**Fig. 2:**
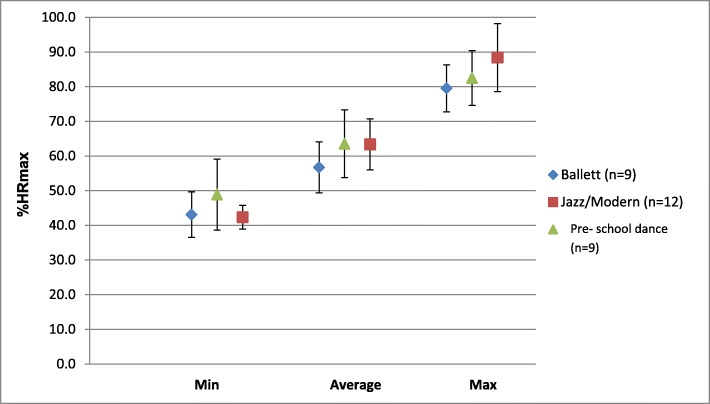
Minimum, average and maximum heart rate intensities of dance teachers (mean values ± standard deviation) in dance units

The increase between minimum and maximum heart rate was about 84% for B, about 109% for J/MD, 69% for pre-school dance and an average of about 88% for all lessons. Between the average and maximum loads during a dance unit, the differences for B and J/MD were about 40% each, for pre-school dance about 30% and for all lessons together about 36% (Table 3, Fig. 2).

**Table 3:** Teaching intensities in dance units (*n* = 31) (according to dance styles with mean value (MW) and associated standard deviation (SD) in percent of the maximum heart rate (% HRmax)

	Minimum intensity (%HRmax)		Average intensity (%HRmax)		Maximum intensity (%HRmax)	
Dance style	mean value	SD	mean value	SD	mean value	SD
**Ballett (n = 9)**	43.1	6.6	56.7	7.4	79.5	6.8
**Jazz/Modern (*n*** **= 12)**	42.3	3.4	63.3	7.3	88.4	9.8
**pre-school dance (*n*** **= 10)**	48.9	10.2	63.6	9.8	82.5	7.9
**All dance styles (n = 31)**	44.7	7.7	61.5	8.8	83.9	9.2

The average heart rates of the entire sample were above 60% of HRmax. The test for mean value differences showed no significant deviations between the minimum, average and maximum cardiovascular loads. However, a statistical tendency (*p* = 0.080) for the comparison within the maximum hourly intensities of the dance styles could be observed. Consideration of the post-doctoral test suggested that there might be a difference between the maximum load of B and J/MD. The average difference was 8.9% with a standard error of 3.9%.

### Differences between lesson sequences

**Fig. 3:**
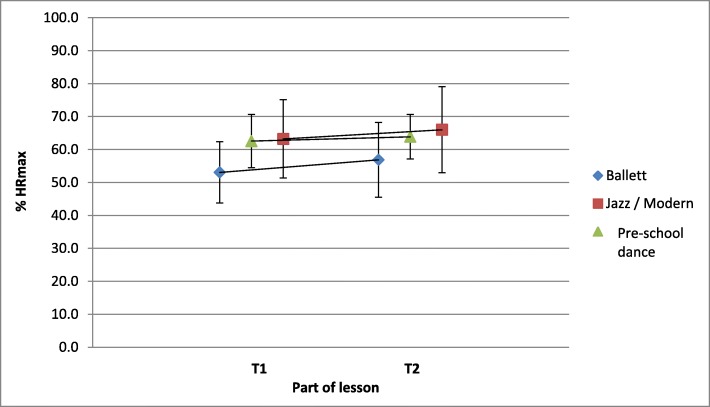
Cardiovascular load of the T1 and T2 sessions in the three dance styles ballet, jazz/modern and pre-school dance (mean value ± standard deviation)

The majority of adult lessons in B, J/MD lasted 90 min. In ballet, one third of the lesson (30 min) was dedicated toT1. This strict subdivision of B could not always be found in J/MD. The pre-school dance lessons attracted attention with partly heterogeneous lesson structures and contents as well as a shorter total duration (between 45 and 60 min). As to the interaction effect between group affiliation and time no significant effect (*p* = 0.557) could be observed. It can be stated that there were only small increases in the average cardiovascular load < 4% HRmax for all three dance styles from the warm-up part to the main part (Table 3, Fig. 3).

### Self-perceptions of lesson loads (RPE)

On average, all dance lessons were evaluated with an RPE value of 11.4 ± 2.1 (Table 4). This corresponds to a “light” feeling of exertion. The individual dance styles were also part of this assessment and did not deviate from each other.

**Table 4:** Values of RPE during teaching units (*n* = 31 units)

Dance style	RPE median	RPE min	PRE max
B (9)	12	8	16
JMD (12)	10,5	8	16
Pre-school dance (10)	11	9	13
Total (31)	11	8	16

Table 5 shows the correlations between the cardiovascular loads within the lessons and the perceived exertion given by means of the RPE scale. A small linear correlation (r = 0.376, *p* = 0.037) for the average heart rates and RPE as well as a mean correlation (r = 0.441, *p* = 0.013) for the maximum heart rate values could be observed for the total sample of DT. The individual evaluation of the individual dance directions revealed no correlations for the B and pre-school dance lessons. However, an average correlation (r = 0.588, *p* = 0.044) for the average heart rates for J/MD could be observed.

**Table 5:** Correlations between RPE and % HRmax of dance teachers in the different dance units (n = 31)

Dance style (n)	% HRmax (mean value ± SD)	RPE (mean value ± SD)	Spearman-correlation (r)	***p*** value
**Minimum intensity**				
B (9)	43.1 ± 6.6	11.3 ± 2.3	0.306	0.423
JMD (12)	42.3 ± 3.4	11.4 ± 2.3	0.528	0.078
pre-school dance (10)	48.9 ± 10.2	11.5 ± 1.4	−0.259	0.469
Total (31)	44.7 ± 7.7	11.4 ± 2.1	0.255	0.166
**Average intensity**				
B (9)	56.7 ± 7.4	11.3 ± 2.3	0.460	0.213
JMD (12)	63.3 ± 7.3	11.4 ± 2.3	0.588	0.044
pre-school dance (10)	63.6 ± 9.8	11.5 ± 1.4	0.304	0.393
Total (31)	61.5 ± 8.8	11.4 ± 2.1	0.376	0.037
**Maximum intensity**				
B (9)	79.5 ± 6.8	11.3 ± 2.3	0.434	0.243
JMD (12)	88.4 ± 9.8	11.4 ± 2.3	0.546	0.067
pre-school dance (10)	82.5 ± 7.9	11.5 ± 1.4	0.278	0.437
Total (31)	83.9 ± 9.2	11.4 ± 2.1	0.441	0.013

## Discussion

Coping with physical and psycho-mental loads is a key function of dance teachers’ work. Although there is a need for research, objective data on workloads are either very limited or of earlier date.

In total, the cardiovascular loads measured for all teaching units with equal distribution were slightly below the results published by Dahlström (1997) [3] The assumption of Dahlström (1997) [3] that teaching children is associated with higher physical strain than teaching advanced dance students could not be proven by this study. However, the results are compatible with the findings of Wanke et al. (2015) [4] where only 26.6% of dance teachers stated their highest physical strain teaching children aging up to 11 years and just as many (29.8%) teaching the 18–35 age group.

Dahlström (1997) [3] found greater increases in cardiovascular stress from warm-up phase T1 to main phase T2 during a lesson than the present study was able to show. One reason could be the different populations of pupils. Teaching experienced adults may allow a more intensive training with fewer breaks in group organization and a consequent drop in heart rate than is the case with beginners and children. This at least is what the heart rate curves of Dahlström (1997) [3] suggest in comparison to this study.

The cardiovascular teaching loads do not represent an adequate training stimulus for increasing basic endurance. The average intensity of a dance lesson of about 61.5% of the HRmax was in the range of a regeneration training and, thus was too low for an increase in endurance performance. Furthermore, the interval-type load design (high HR peaks and consequently high HR drops) did not correspond to the necessary long-term method (constant HR rates with low fluctuations) [18]. This becomes particularly clear through HR drops in areas of rest frequencies and HR ups in areas of maximum performance. In addition, the high-intensity range (90–95% HRmax) is reached too sporadically and too briefly during teaching to lead to an increase in cardiovascular fitness in the sense of high-intensity interval training (HIT) [19]. However, a good basic endurance capacity is an important aspect of prevention [20–22]. Therefore, additional training should be considered.

### Differences between the dance styles

It could be observed in most ballet lessons that a good division into a barre part (including supporting exercises) and a center work part (with adagio, jumps and turns) was possible. In purely quantitative terms, the barre part in this study was somewhat shorter than that stated in Schantz & Astrand’s (1984) [17] with about 40% and in Dahlström’s (1997) [3] study. The heterogeneous lessons in J/MD and pre-school dance could not show such a clear quantitative distinction. As to the pre-school dance, statements by Stinson (1988) [23] can be confirmed, who determined different teaching methods depending on the mood, behavior patterns of the children and other group-specific characteristics. These factors demand flexible actions by the dance teacher and result in significant differences between the individual pre-school dance lessons.

As to the minimal cardiovascular load, no differences were found between the dance styles., Like Dahlström’s findings (1997) [3], this study showed no differences between the dance styles for the medium and maximum loads. However, first tendencies between maximum stress in ballet and J/MD were observed. The difference between J/MD and the three other dance styles (ballet, character dance and modern dance), which Dahlström (1997) [3] found for dancers, could also apply to dance teachers. A larger sample could possibly clarify this issue.

### RPE to assess physical work loads

For the assessment of one’s own cardiovascular load in the course of a lesson significant correlations could be observed for the mean and maximum heart rate intensities to the perception of exertion, Originally intended to assess the average loads, the Borg scale seems to be better suited for assessing the maximum load regardless of the dance style. Even though all correlation coefficients were lower than stated by Chen et al. (2002) [10] and only medium correlations could be observed, the subgroup analysis in the field of J/MD showed a correlation that was only slightly lower. Thus, these results are also somewhat lower than those of Surgenor et al. (2019) [24], who found high construct validity for RPEs. Based on the WHO (2010) [25] health recommendations for physical activity, which are 150 min per week of moderate intensity (corresponds to a 5 or 6 on a scale of 0–10) or alternatively 75 min per week of strenuous intensity (corresponds to a 7 or 8 on a scale of 0–10) for people between 18 and 64 years of age, the dance teachers’ teaching workload is significantly higher. In contrast, the individual average cardiovascular stress appears to be relatively low. In addition, the recorded RPE estimates of an average of 11.4 are at the lower limit of moderate stress (Norton et al., 2010). This leads to the assumption that, despite a high overall extent, no endurance-effective training stimulus is achieved through occupational activity [26]. .Considering the high amount of lessons and regenerative breaks seemingly short [5] as well as the realization that injury must be avoided as far as possible, a guided additional endurance training combined with a preliminary sports medical examination seems sensible in order to ensure coping with the permanent workload, prophylactic protection against injuries and faster regeneration after injuries occurring in the passive musculoskeletal system as described [6, 7, 27, 28]. At that, the interval-like load character of many dance lessons with partly high workload peaks, which are not really striking when looking at the average values, should be taken into account. Sudden high movement intensities without a physical warm-up prior to them could pose an existential risk due to the injury hazard [6]. An assessment of an entire work day [28] as well as of the weeks and a monthly structure would be advantageous to better understand the loads to breaks ratio. In addition, leisure activities could be recorded in order to highlight possible compensatory hobbies or other activities involving physical exertion. The mental component of dance teachers’ profession is not to be neglected. According to the present study, teaching children is associated with lower physical stress, but DTstated a significantly higher psychological stress [4].

The findings of Bogaert et al. (2014) [29] showed that high physical activity does not necessarily lead to high self-perception of mental and physical health. The DTs showed a high physical activity, but according to Wanke et al. (2015) [4] also a high feeling of load. Whether the same connections between psychosocial load, the occurrence of injuries and the duration of injuries as found in dancers [30, 31] also applies to DTs have yet to be investigated. It is also unclear what influence the permanent support in the pre-school dance lessons and whether mostly unfavourable body position in which the dance teachers remain, affect the musculoskeletal system.

In order to obtain more valid estimates of physical exertion with the RPE scale, a longer exercise phase to familiarize with it would possibly be advantageous for subjects. In addition, in future diagnostic research, the hypothesis of dichotomy of the perception of effort on the basis of Pandolf (1981) [32] is suggested be able to assess local and general RPE separately. Furthermore, a survey instrument for measuring mental stress would be conceivable. This would make a maximization of the correlation between heart rate and general RPE possible by partializing these RPE components because it can be assumed that the focus is mainly on the female pupils and less on the self-perception of one’s own demands.

All in all, the present study makes it clear that an assessment of cardiovascular load on the basis of heart rate recording of different teaching units of different dance styles allows only initial rough assessments. In order to obtain as precise information as possible about the cardiovascular demands, further investigations with larger numbers of test persons and perhaps other measuring methods are needed. The recording of HR could be combined with video-based and accelerometric methods, in order to discriminate physically active phases from pauses of movement.

Nevertheless, the results show that maximum cardiovascular load was achieved in some cases and that there were clear fluctuations between the individual teaching units. Significant differences between the dance styles, however, could not be observed.

As to the J/MD, the use of RPE would be conceivable in order to provide a rough estimate of cardiovascular load, especially since the effort involved in collecting data is extremely low. Furthermore, our study suggests the strong assumption that teaching in the dance classes does not usually provide an adequate stimulus in the sense of basic endurance training to increase aerobic capacity.

Balancing training in the area of basic endurance makes sense if one considers the high number and the interval-like load character of teaching units. In addition, further studies on the correlation between physical performance and acute or chronic injuries will be important in future. The evaluation of further health-related data, such as nutritional behaviour and knowledge of a healthy lifestyle, both important as basis for one’s own actions and for communicating them to the dance students, is still pending [32, 33]. In addition, attention should be paid to explicit psychological investigations of the mental load of the occupational group of dance teachers. The analysis of lesson plans of weekly or monthly rhythms in the sense of a mesocyclic view would be advantageous in order to be able to better record the timing of teaching units and thus the rhythm between regenerative breaks and workloads.

### Limitations

The main limitation of the study is certainly the measurement of the heart rate itself as a load indicator. Redding et al. (2004) [34] and Wyon et al. (2004) [35] emphasize that cardiovascular drifts can occur during intermittent loads accompanied by increased heart rates during pauses. Misjudgements of the load profiles would be the consequence without further participating observations. In addition, the decrease in heart rate strongly depends on the aerobic capacity of the respective test subject.

It is also to be considered that the change in heart rate depends on a number of influencing factors. Besides the purely motor activity, the hydration status [36], music [37] or mental load [38] for example, have an effect on the heart rate. Since such parameters were not explicitly recorded, the heart rate as the sole parameter is at least to be interpreted with caution when assessing cardiovascular load, as already worded by Wyon (2009) [39].

Furthermore, the authors are aware that different methods for estimating relative load intensities (% HRR, VO2R, etc.) are discussed [40]. An attempt was made in this study to measure HRmax as accurately as possible by spiroergometry and to use it as a baseline for load relativization.

Although the average percentage of cardiovascular load did not show any major differences, the heart rate curves illustrate other stress curves that indicate the individual character of the individual dance styles and differences in the target group to be taught. A further differentiation of the lessons in B and J/MD for young people, young adults and older adults and a clear distinction between beginner and advanced lessons would be desirable for the future. However, this classification was not possible due to the few subjects who did not usually teach in all age and level groups. In general, the rather small sample only permits initial rough assessments in the sense of a pilot study and does not allow any general validity.

For the comparison between the warm-up phase and the main phase, the time division of the lessons into T1 and T2 certainly only represents a compromise consisting of qualitative and quantitative points of view, which do not always correspond to the real temporal selectivity of a teaching process and may thus lead to distortions. Measurement artifacts were caused by the application of the transmitter, the degree of moisture between transmitter and subject’s skin and the electrical contacts between transmitter belt and technical transmitter unit.

## Conclusion

Cardiovascular load in dance classes is intermittent with strong individual fluctuations to be investigated further by a more precise measurement method. Furthermore, the self-assessment of these teaching demands on the basis of RPE is only partially and roughly possible. A higher cardiopulmonary performance would be desirable for persons without facilitating work equipment. In the future, studies with a stronger consideration of the connections between teaching load, occurrence of illness and injury as well as the general state of health and health behaviour are desirable.

## Data Availability

The dataset supporting the conclusions of this article is included within the article.
